# Antioxidant and Neuroprotective Activity of Extra Virgin Olive Oil Extracts Obtained from Quercetano Cultivar Trees Grown in Different Areas of the Tuscany Region (Italy)

**DOI:** 10.3390/antiox10030421

**Published:** 2021-03-10

**Authors:** Maria Cristina Barbalace, Lorenzo Zallocco, Daniela Beghelli, Maurizio Ronci, Serena Scortichini, Maria Digiacomo, Marco Macchia, Maria Rosa Mazzoni, Dennis Fiorini, Antonio Lucacchini, Silvana Hrelia, Laura Giusti, Cristina Angeloni

**Affiliations:** 1 Department for Life Quality Studies, Alma Mater Studiorum-University of Bologna, 40126 Bologna, Italy; maria.barbalace2@unibo.it (M.C.B.); silvana.hrelia@unibo.it (S.H.); 2 Department of Pharmacy, University of Pisa, 56126 Pisa, Italy; l.zallocco@gmail.com (L.Z.); maria.digiacomo@unipi.it (M.D.); marco.macchia@unipi.it (M.M.); maria.mazzoni@unipi.it (M.R.M.); 3 School of Biosciences and Veterinary Medicine, University of Camerino, 62032 Camerino, Italy; daniela.beghelli@unicam.it; 4 Department of Pharmacy, University “G. d’Annunzio” of Chieti-Pescara, Via dei Vestini, 66100 Chieti, Italy; maurizio.ronci@unich.it; 5 Center for Advanced Studies and Technology (CAST), University “G.d’Annunzio” of Chieti-Pescara, Via dei Vestini, 66100 Chieti, Italy; 6 School of Science and Technology, Chemistry Division, University of Camerino, V.S. Agostino 1, 62032 Camerino, Italy; serena.scortichini@unicam.it (S.S.); dennis.fiorini@unicam.it (D.F.); 7 Department of Clinical and Experimental Medicine, University of Pisa, 56126 Pisa, Italy; antonio.lucacchini@gmail.com; 8 School of Pharmacy, University of Camerino, 62032 Camerino, Italy; laura.giusti@unicam.it

**Keywords:** olive oil, oxidative stress, antioxidants, phenols, neuroprotection, neurotrophins, proteomics

## Abstract

Neurodegenerative diseases are driven by several mechanisms such as inflammation, abnormal protein aggregation, excitotoxicity, mitochondrial dysfunction and oxidative stress. So far, no therapeutic strategies are available for neurodegenerative diseases and in recent years the research is focusing on bioactive molecules present in food. In particular, extra-virgin olive oil (EVOO) phenols have been associated to neuroprotection. In this study, we investigated the potential antioxidant and neuroprotective activity of two different EVOO extracts obtained from Quercetano cultivar trees grown in two different areas (plain and hill) of the Tuscany region (Italy). The different geographical origin of the orchards influenced phenol composition. Plain extract presented a higher content of phenyl ethyl alcohols, cinnammic acids, oleacein, oleocanthal and flavones; meanwhile, hill extract was richer in lignans. Hill extract was more effective in protecting differentiated SH-SY5Y cells from peroxide stress thanks to a marked upregulation of the antioxidant enzymes heme oxygenase 1, NADPH quinone oxidoreductase 1, thioredoxin Reductase 1 and glutathione reductase. Proteomic analysis revealed that hill extract plays a role in the regulation of proteins involved in neuronal plasticity and activation of neurotrophic factors such as BDNF. In conclusion, these data demonstrate that EVOOs can have important neuroprotective activities, but these effects are strictly related to their specific phenol composition.

## 1. Introduction

In the “era” of the economic prosperity, the non-communicable diseases such as cardiovascular diseases, neurodegenerative diseases, cancer, obesity and diabetes, are collectively accountable for almost 70% of all deaths worldwide [1].

The complexity of these diseases makes it difficult to fight them with single-target molecules. Indeed, the research is shifting its strengths on multitarget compounds [2,3] and in recent years it is focusing on bioactive molecules naturally present in food as potential “weapons” against chronic degenerative diseases. On the base of these premises, nutrition could represent a key factor of lifestyle for counteracting the development and progression of such pathologies. In particular, the Mediterranean diet, rich in vegetables, cereals, fruits, legumes and monounsaturated fatty acids mainly derived from olive oil, has been associated with a reduced incidence of neurodegenerative diseases and enhanced cognitive performance [4,5].

Neurodegenerative diseases represent an increasingly public health problem, especially in the aging population. These pathologies are multifactorial non communicable diseases driven by several but linked mechanisms such as inflammation, abnormal protein aggregation, excitotoxicity, oxidative stress and mitochondrial dysfunction [6,7,8,9,10]. In addition, the accumulation of proteins and lipids modified by the excessive production of reactive oxygen species (ROS) leads to a self-feeding loop [11]. Oxidative stress consists in an imbalance condition where the production of reactive species (mainly ROS) exceeds their detoxification, leading to an abnormal accumulation of radical species and oxidative damages. Furthermore, oxidative stress triggers and is triggered by mitochondrial dysfunction [12]. Stated the key role played by mitochondria in energy metabolism and modulation of redox homeostasis, their dysfunction might contribute to the pathogenesis of neurodegeneration. Due to its high oxygen requirements, relative poorness of antioxidant defenses, elevated levels of unsaturated fatty acids and abundance in iron content, the brain is particularly sensible to oxidative stress [13,14]. Consequently, neurons are prone to oxidative injury and the physiological process of aging together with the aging-associated diseases share the promotion of a redox imbalance [15]. Of note, neurons are cells characterized by limited self-renewal, implying that damages can accumulate over time [13]. Regardless of how oxidative stress is caused, when encountered, cells counteract the damaging effect of this condition by activating the endogenous antioxidant defense system, which is unfortunately compromised in the context of neurodegeneration. Extensive studies during the past decade have proven the notion that the transcription factor NF-E2-related factor 2 (Nrf2) is an essential element for up-regulating the antioxidant defense system [16,17,18].

In addition, neuronal growth and survival are ensured by the neurotrophic signaling pathway and alteration of specific neurotrophic factors leads to brain degeneration [19]. In particular, the brain-derived neurotrophic factor (BDNF) is essential for the survival and normal functioning of mature neurons and its level are markedly depressed in neurodegenerative diseases [20,21,22]. These relationships suggest the BDNF and the Nrf2 signaling pathway are potential targets for supporting neuronal survival and regeneration of impaired neuronal structures and synaptic connectivity.

Unfortunately, to date, no therapeutic strategies are available for neurodegenerative diseases.

It has been suggested that the positive role of the Mediterranean diet is probably related to the presence of a huge quantity and different phenols [23,24]. One of the main source of phenols from the Mediterranean diet is the consumption of extra-virgin olive oil (EVOO) in amounts going from 30 to 50 g/day [25], representing the principle fat consumed in the Mediterranean diet [26]. In vitro and in vivo studies demonstrated preventive and protective properties of EVOO against neurodegeneration. The major polar phenolic compounds present in EVOO such as oleacein (dialdehydic form of decarboxymethyl elenolic acid linked to hydroxytyrosol, 3,4-DHPEA-EDA), oleuropein aglycone isomer (3,4-DHPEA-EA), oleocanthal (the dialdehydic form of decarbox-ymethylelenolic acid linked to tyrosol, p-HPEA-EDA), ligstroside aglycon (p-HPEA-EA), hydroxytyrosol (3,4-DHPEA), tyrosol (p-HPEA), have been demonstrated to possess anti-inflammatory [27], anti-microbial and anti-viral activities [28], to counteract oxidative stress and modulate survival signaling pathways [29]. Many factors can influence EVOO phenolic composition and concentration including cultivars, climate conditions, ripening stage of the olives but also olive oil production processes and storage [30,31]. Therefore, the definition of an exact phenolic content is hard and referring to different studies the amount could range from 200 to 1000 mg/kg [28,30,32,33,34,35]. Furthermore, the specific phenolic pattern of different EVOOs could influence their biological activity such as neuroprotection.

In this study, we investigated the potential antioxidant and neuroprotective effects of two different EVOO extracts obtained from Quercetano cultivar trees grown in two different areas of the Tuscany region (Italy). In particular, we characterized the extracts by HPLC-DAD/MS and evaluated their neuroprotective activity against H_2_O_2_-induced oxidative stress in differentiated SH-SY5Y cells. Moreover, proteomics combined with pathways analyses, was used to investigate the molecular events related to the protective effects and to gain insight into the underlying mechanisms of neuroprotection.

## 2. Materials and Methods

### 2.1. Chemicals

3-(4,5-dimethylthiazol-2-yl)-2,5diphenyl-tetrazolium bromide (MTT), 2,7-dichlorodihydrofluorescein diacetate (DCFH-DA), H_2_O_2_, dimethyl sulfoxide (DMSO), monochlorobimane (MCB), Phosphate Buffered Saline (PBS), Dulbecco’s modified Eagle’s medium (DMEM), penicillin/streptomycin, primers listed in Table 1, all trans retinoic acid (RA), Lactate Dehydrogenase Activity Assay Kit and all other chemicals of the highest analytical grade were purchased from Sigma-Aldrich (Milano, Italy). Fetal bovine serum (FBS) was purchased from Euroclone (Milano, Italy).

### 2.2. EVOO Sample Extraction

EVOO samples were obtained from the fruits of the olive cultivar Quercetano (*Olea europaea* L.) which were picked at the same stage of maturity during the same month season 2019 in two olive groves located at different altitude (hill, plain) and with some different soil type (clay, silt, sandy) in Tuscany region (Italy). The phytoextracts of hill and plain EVOOs were prepared essentially as previously described [36,37]. Briefly, EVOO (3 g) was mixed with n-hexane (12 mL) and acetonitrile (15 mL). The resulting mixture was homogenized using a vortex mixer and centrifuged at 4000 rpm for 5 min, at 25 °C. Then, the acetonitrile phase was collected and evaporated under reduced pressure, to obtain the phytoextracts.

### 2.3. Analysis of Phenolic Substances

The extracts to be analyzed were weighted (7–19 mg) and then each one was dissolved in 0.46 mL methanol/water solution (6:4 *v/v*), added with 0.04 mL of internal standard solution (syringic acid in methanol, at 100 mg/L) and with 0.5 mL of hexane to remove possible triacylglycerols left. The biphasic system was shaken with the help of a vortex for 2 min, then centrifuged and then hexane upper phase removed. The hydro-methanolic solution was filtered and then analyzed by HPLC-DAD/MS with the condition reported in Ricciutelli et al., 2017 [38]. Quantification of the phenolic substances hydroxytyrosol, tyrosol, ferulic acid, p-coumaric acid, vanillic acid, 3,4-DHPEA-EDA, p-HPEA-EDA, 3,4-DHPEA-EA, p-HPEA-EA, pinoresinol, acetoxypinoresinol, luteolin, apigenin was performed according to [39]. Briefly, HPLC-DAD analysis, that was used for the quantification, was performed monitoring different wavelengths: 260 nm for vanillic acid, 280 nm for tyrosol, hydroxytyrosol, secoiridoids derivatives (namely 3,4-DHPEA-EDA, p-HPEA-EDA, 3,4-DHPEA-EA, p-HPEA-EA), pinoresinol, acetoxypinoresinol and syringic acid; 310 nm for p-coumaric acid, 325 nm for ferulic acid, 338 nm for apigenin and 350 nm for luteolin. Secoiridoid derivatives were quantified using tyrosol calibration curve. Mass spectrometer was used only for confirmation of the identity of the analytes.

### 2.4. Cell Culture and Treatments

The SH-SY5Y human neuroblastoma cell line was obtained from Sigma-Aldrich (Milan, Italy). Cells were grown in DMEM supplemented with 10% (*v/v*) FBS, 2 mM glutamine, 50 U/mL of penicillin and 50 μg/mL of streptomycin and maintained at 37 °C in a humidified incubator with 5% CO_2_ as reported in [40]. Cell differentiation was induced reducing serum levels of the medium to 1% with 10 µM of RA for seven days prior to treatments. Differentiated SH-SY5Y were treated with plain and hill extracts for 24 h. Extracts were dissolved in DMSO. The control group was treated with an equivalent volume of the vehicle alone. Oxidative stress was induced exposing cells to 700 µM H_2_O_2_ for different times depending on the assay.

### 2.5. MTT and Lactate Dehydrogenase Activity Assays

Cells were treated with different concentrations of plain and hill extracts (1 µg/mL–500 µg/mL) for 24 h. The induction of oxidative stress was achieved with 700 µM of H_2_O_2_ for 1 h. Cell viability was evaluated by measuring formazan formation as previously reported [41]. Cells were incubated with 0.5 mg/mL of MTT solution for 1 h at 37 °C. After incubation, MTT was removed and 100 µL of DMSO were added, the absorbance was recorded at λ = 595 nm using a microplate spectrophotometer (VICTOR3 V Multilabel Counter; PerkinElmer, Wellesley, MA, USA). Lactate dehydrogenase (LDH) activity was evaluated in the culture medium and the test was performed using the Lactate Dehydrogenase Activity Assay Kit according to the manufacturer’s instructions.

### 2.6. Intracellular ROS Production Assay

The fluorescent probe DCFH-DA was used to monitor the production of intracellular reactive oxygen species [42]. Differentiated SH-SY5Y were treated with different concentrations of plain and hill extracts for 24 h and then incubated with 10 µg/mL DCFH-DA in DMEM w/o FBS for 30 min. After DCFH-DA removal, cells were incubated with 200 µM H_2_O_2_ for 15 min. Cell fluorescence was measured using 485 nm excitation and 535 nm emission with a microplate spectrofluorometer (VICTOR3 V Multilabel Counter).

### 2.7. Intracellular GSH Levels Assay

The levels of reduced glutathione (GSH) were evaluated by a fluorometric assay, using the fluorescent probe MCB [43]. Briefly, after treatments, differentiated SH-SY5Y were incubated with 50 µM MCB in serum-free medium for 30 min at 37 °C. After incubation, fluorescence was measured at 355 nm (excitation) and 460 nm (emission) with a microplate spectrofluorometer (VICTOR3 V Multilabel Counter; PerkinElmer).

### 2.8. RNA Extraction

Total RNA was extracted using RNeasy Mini Kit (QIAGEN GmbH, Hilden, Germany), following the manufacturer’s protocol. The yield and purity of the RNA were measured using NanoVue Spectrophotometer (GE Healthcare, Milano, Italy).

### 2.9. Analysis of mRNA Levels by Reverse Transcriptase Polymerase Chain Reaction

cDNA was obtained by reverse transcribing mRNA starting from 1 μg of total RNA using iScript cDNA Synthesis Kit (BIO-RAD, Hercules, CA, USA), following the manufacturer’s protocol. The subsequent polymerase chain reaction (PCR) was performed in a total volume of 10 μL containing 2.5 μL (12.5 ng) of cDNA, 5 μL SsoAdvanced Universal SYBR Green Supermix (BIO-RAD) and 0.5 μL (500 nM) of each primer (Sigma-Aldrich). The primers used are reported in Table 1, 18S rRNA was used as reference gene.

### 2.10. Proteomic Analysis

For proteomic analysis, differentiated SH-SY5Y cells were treated with plain and hill extracts (10 μg/mL) for 24 h as described above. At the end of treatments, cells were collected and washed with PBS. After centrifugation (1000× *g* for 5 min), the resulting pellets were immediately frozen and stored at −80 °C until use. For proteomic studies, each condition was performed in triplicate.

Cell pellets were resuspended in rehydration solution [44] and protein contents of resulting protein extracts were measured with an RC-DC Protein Assay from Bio-Rad.

The 2DE was carried out as previously described [45]. Briefly, 200 µg of proteins were filled up to 450 μL in rehydration solution. Immobiline Dry-Strips (GE Health Care Europe; Uppsala, Sweden); 18 cm, nonlinear gradient (pH 3–10) were rehydrated overnight in the sample and then transferred to the Ettan IPGphor Cup Loading Manifold (GE Healthcare) for isoelectrofocusing (IEF). The second dimension (Sodium Dodecyl Sulphate-Polyacrylamide Gel Electrophoresis; SDS-PAGE) was carried out by transferring the proteins to 12% polyacrylamide, running at 16 mA per gel and 10 °C for about 16 h, using the Protean^®^ Plus Dodeca Cell (Bio-Rad). The gels were stained with Ruthenium II tris (bathophenanthroline disulfonate) tetrasodium salt (Cyanagen Srl, Bologna, Italy) (RuBP). ImageQuant LAS4010 (GE Health Care) was used for the acquisition of images. The analysis of images was performed using Same Spot (v4.1, TotalLab; Newcastle Upon Tyne, UK) software. The spot volume ratios among the three different conditions (Control, hill, plain) were calculated using the average spot normalized volume of the three biological replicates performed in duplicate. The software included statistical analysis calculations.

### 2.11. Spot Digestion and Protein Identification

The gel pieces were trypsin digested and analyzed by LC-MS as previously described [46]. Each digested spot sample was analyzed by LC-MS/MS using a Proxeon EASY-nLCII (Thermo Fisher Scientific, Milan, Italy) chromatographic system coupled to a Maxis HD UHR-TOF (Bruker Daltonics GmbH, Bremen, Germany) mass spectrometer. The raw data were processed using PEAKS Studio v7.5 software (Bioinformatic Solutions Inc, Waterloo, ON, Canada) using the function “correct precursor only”. The mass lists were searched against the nextprot database including isoforms (version as of June 2017; 42,151 entries) using 10 ppm and 0.05 Da as the highest error tolerances for parent and fragment ions, respectively. Carbamidomethylation of cysteines was selected as fixed modification and oxidation of methionines and deamidation of asparagine and glutamine as variable modifications allowing two missed cleavages.

### 2.12. Bioinformatic Analysis

For gene ontology analysis, including differential molecular function and biological processes, PANTHER software (Protein Analysis Through Evolutionary Relationships; http://www.pantherdb.org/genes/batchIdSearch.jsp (accessed 3 January 2021), was used to classify genes into distinct categories of molecular functions and biological processes. Proteins differentially expressed obtained both from hill vs Control and plain vs Control comparisons were functionally analyzed using QIAGEN’s Ingenuity Pathway Analysis (IPA, QIAGEN Redwood City, USA, www.qiagen.com/ingenuity, Build version: 321501M Content version: 21249400, accessed 15 January 2021) with the aim to determine the predominant canonical pathways and interaction network involved. Swiss-Prot accession numbers and official gene symbols were inserted into the software along with corresponding comparison ratios and *p* values. Based on known associations in the literature, canonical pathways associated with differentially expressed proteins were defined. A comparison of the different analyses was created and the upstream regulators whose activity appears to change in a significant manner according to the activation z-score value were shown. Finally, the impact of activated or inhibited regulators on downstream functions and diseases were investigated. The use of an algorithm allowed to merge upstream and downstream results from the upstream regulator, through one or more iterations. The networks were merged only if the overlap of protein targets was possible and of statistical significance (Fisher’s Exact Test). Higher scoring hypotheses were those with more consistent causal paths represented by a high Consistency Score.

### 2.13. Western Blot Analysis

Western blot (WB) was performed in order to validate peculiar increase of expression of both DPYSL2 and BDNF found in hill with respect to plain and Control samples with 2-DE and RT-PCR, respectively. Aliquots of protein samples (5 μg for DPYSL2 and 50 μg for BDNF) were mixed with Laemmli solution, run in 4–15% polyacrylamide gels (Mini-PROTEAN^®^ Precast Gels, Biorad, Hercules, CA, USA) using a mini-Protean Tetracell (Biorad, Hercules, CA, USA) and transferred onto nitrocellulose membranes (0.2 μm) using a Trans-Blot Turbo transfer system (Biorad) as previously described [45]. Anti-DPYSL2 (Cell Signaling Technology, Beverly, MA, USA) and anti-BDNF (Genetex, Irvine, CA, USA) antibodies were used at 1:1000 dilution. Moreover, an anti-β-actin (Merck KGaA, Darmstadt, Germany), was used for internal normalization. HRP-goat anti-rabbit secondary antibody was used at 1:10,000 dilution. Immunoblots were developed using the enhanced chemiluminescence detection system (ECL). The chemiluminescent images were acquired using LAS4010 (GE Health Care Europe, Upsala, Sweden). The immunoreactive specific bands were quantified using Image Quant-L software.

### 2.14. Statistical Analysis.

All analyses were performed at least in triplicate and values were expressed as mean ± standard error. In experiments with SH-SY5Y cell cultures, one-way ANOVA was used to compare differences among groups followed by Dunnett’s or Bonferroni’s test (Prism 5; GraphPad Software, San Diego, CA, USA). Differences at the level *p* < 0.05 were considered statistically significant. In 2DE experiments, a comparison among the different treatments was performed. The significance of the differences of normalized volume for each spot was calculated by the software Same Spot including the analysis of variance (ANOVA test). The protein spots significantly differentially expressed were cut out from the gel and identified by LC-MS analysis.

## 3. Results

### 3.1. Phenolic Content

The soil characteristic of the hill and plain orchards are reported in Table 2. Interestingly, the two soils were very different in terms of silt, sandy, pH and mineral composition.

The analysis of phenolic components of EVOOs extracts from the hill and plain orchards shows a similar qualitative composition, but a different quantitative composition. In particular, the total phenol content is higher in the plain than in the hill extract (Table 3). Moreover, phenyl ethyl alcohols (tyrosol, hydroxytyrosol), oleacein (3,4-DHPEA-EDA), oleocanthal (p-HPEA-EDA), cinnammic acids (p-coumaric acid, ferulic acid), flavones (luteolin, apigenin) are higher in the plain extract, vice versa lignans (pinoresinol, acetoxypinoresinol), oleuropein aglycon isomer (3,4-DHPEA-EA) and ligstroside aglycon (p-HPEA-EA) are higher in hill extract.

### 3.2. EVOO Extracts—Mediated Neuroprotection against Oxidative Damage

**Figure 1:**
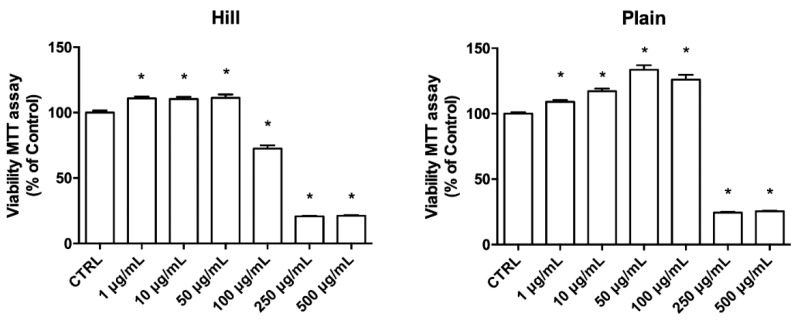
Viability of differentiated SH-SY5Y treated with EVOO extracts. Cells were treated with increasing concentrations (1 to 500 µg/mL) of EVOO extracts for 24 h and the cellular viability was measured by MTT assay as described in Materials and Methods. Each bar represents means ± SEM of at least three independent experiments. Data were analyzed by one-way ANOVA followed by Dunnett’s test. * *p* < 0.05 with respect to CTRL.

To assess the potential cytotoxicity of the EVOO extracts, differentiated SH-SY5Y cells were exposed to increasing concentrations of the extracts for 24 h (Figure 1). Treatment with the hill extract led to a significant decrease of cell viability in respect to control cells at concentrations higher than 50 µg/mL, meanwhile treatment with the plain extract significantly reduced cell viability at concentrations higher than 100 µg/mL. Interestingly, both extracts significantly increased cell viability at the lowest concentrations.

**Figure 2:**
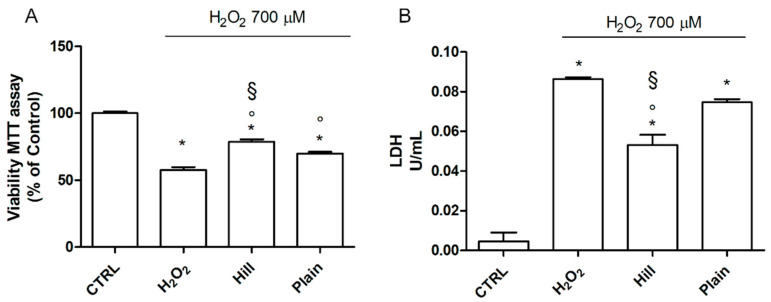
Neuroprotective activity of EVOO extracts against H_2_O_2_ in SH-SY5Y cells. Cells were treated with 10 µg/mL of the EVOO extracts for 24 h and then exposed to H_2_O_2_. (**A**) Cell viability was measured by MTT assay and (**B**) LDH was measured as LDH activity in in the culture medium as described in Materials and Methods. Each bar represents means ± SEM of at least three independent experiments. Data were analyzed by one-way ANOVA followed by Bonferroni’s test. * *p* < 0.05 with respect to CTRL; ° *p* < 0.05 with respect to H_2_O_2_; § *p* < 0.05 with respect to plain.

To assess the neuroprotective activity of the extracts, SH-SY5Y cell were pretreated with 10 µg/mL of the extracts and after 24 h were exposed to H_2_O_2_ to induce oxidative stress (Figure 2). This extract concentration was selected as it is one order of magnitude lower than the toxic concentration and can be considered safe. Peroxide induced a strong and significant reduction of cell viability in respect to control cells, meanwhile both hill and plain extracts were able to significantly increase cell viability in respect to cells exposed to H_2_O_2_ (Figure 2A). Of note, the hill extract significantly increased cell viability in respect to the plain extract. Concerning LDH release, a marker of no-specific cell damage, H_2_O_2_ triggered a strong and significant increase of LDH release in respect to control cells (Figure 2B). Only the hill extract was able to significantly reduce the activity of LDH in the culture medium in respect to H_2_O_2_ exposed cells, confirming the higher antioxidant activity of the hill in respect to the plain extract.

### 3.3. Antioxidant Properties of Hill and Plain Extracts

**Figure 3:**
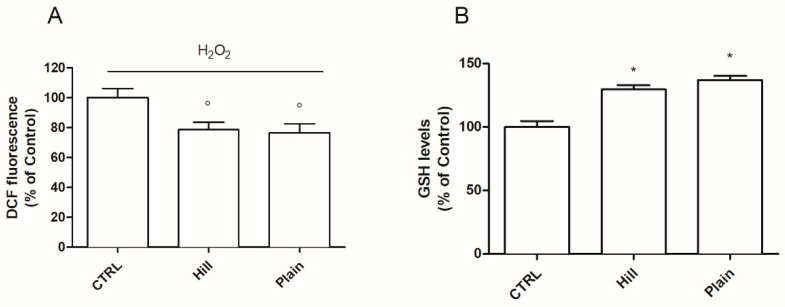
Antioxidant capacity of EVOO extracts. Cells were treated with 10 µg/mL of hill and plain extracts for 24 h (**A**) The intracellular ROS levels were measured with the peroxide-sensitive probe DCFH-DA as reported in Materials and Methods. Data are expressed as percentage of H_2_O_2_. (**B**) The intracellular GSH levels were evaluated using the fluorescent probe monochloro bimane (MCB) as described in Materials and Methods. Data are expressed as percentage of control (CTRL). Each bar represents means ± SEM of at least three independent experiments. Data were analyzed by one-way ANOVA followed by Dunnett’s test or Bonferroni’s test. * *p* < 0.05 with respect to CTRL; ° *p* < 0.05 with respect to H_2_O_2_.

The potential antioxidant activity of hill and plain extracts was investigated through the assessment of intracellular ROS and reduced glutathione (GSH) levels, the main endogenous intracellular antioxidant [47] (Figure 3).

The ability of plain and hill extracts to counteract H_2_O_2_-induced ROS production was evaluated by the DCFH-DA assay. Differentiated SH-SY5Y cells were treated with 10 µg/mL of hill and plain extracts for 24 h and then exposed to H_2_O_2_. As reported in Figure 3A, both extracts significantly reduced ROS levels in respect to H_2_O_2_ treated cells. Then, SH-SY5Y cells were treated with EVOO extracts for 24 h and GSH basal levels were measured by the MCB assay (Figure 3B). In agreement with the previous data, both extracts were able to significantly increase GSH levels compared to control cells.

### 3.4. Modulation of Antioxidant and Pro-Survival Genes by Plain and Hill Extracts

Since the hill extract demonstrated a higher neuroprotective activity against oxidative stress compared to the plain extract, we next drilled down the mechanism underlying such protection.

**Figure 4:**
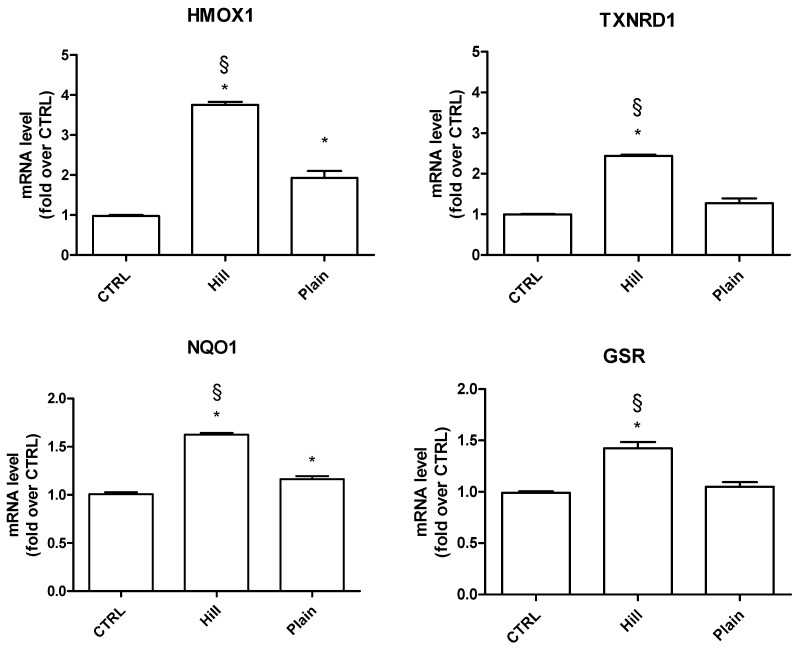
Expression of antioxidant enzymes in SH-SY5Y cells treated with the extracts. Cells were treated with hill and plain extracts (10 µg/mL) for 6 h. Real time-PCR was performed to detect HMOX1, TXNRD1, NQO1 and GSR mRNA levels. Data are expressed as relative abundance compared to untreated cells. Each bar represents mean ± SEM of three independent experiments. Data were analyzed with a one-way ANOVA followed by the Bonferroni’s test. * *p* < 0.05 vs. CTRL; § *p* < 0.05 vs. plain.

To this purpose, we measured the expression of Nrf2-driven antioxidant genes, heme oxygenase 1 (HMOX1), thioredoxin reductase 1 (TXNRD1), NADPH quinone oxidoreductase 1 (NQO1) and glutathione reductase (GSR) in SH-SY5Y cells after treatment with the extracts (Figure 4). The plain extract was able to significantly increase the expression of HMOX1 and NQO1 genes in respect to control cells. On the other hand, the hill extract not only significantly upregulated all four of the genes in respect to control, but it also significantly increased their expression in respect to the plain extract, suggesting a higher ability of the hill extract to enhance the antioxidant defense system.

**Figure 5:**
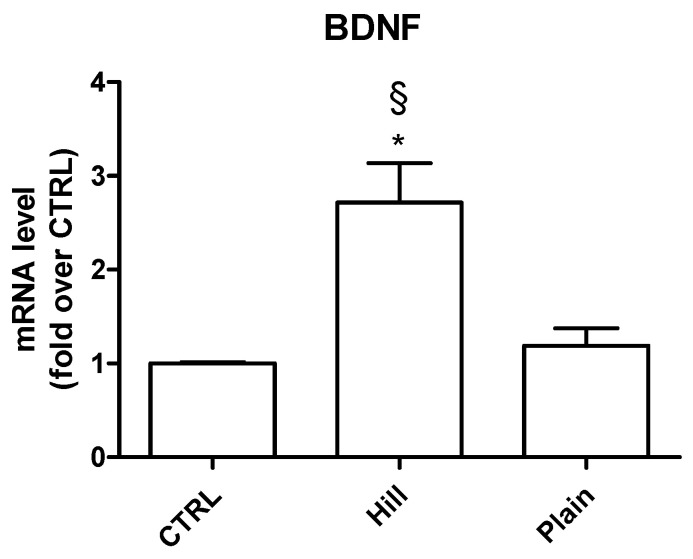
Expression of BDNF in SH-SY5Y cells treated with the extracts. Cells were treated with hill and plain extracts (10 µg/mL) for 24 h. Real time-PCR was performed to detect BDNF mRNA levels. Data are expressed as relative abundance compared to untreated cells. Each bar represents mean ± SEM of three independent experiments. Data were analyzed with one-way ANOVA followed by the Bonferroni’s test. * *p* < 0.05 vs. CTRL; § *p* < 0.05 vs. plain.

Since neurotrophins, such as BDNF, play a fundamental role in neuronal survival [19], we investigated the effect of the treatment with the extracts on BDNF gene expression (Figure 5). Once again, the hill extract demonstrated a higher bioactivity significantly upregulating BDNF gene in respect both to control and plain extract treated cells. The plain extract did not influence the expression of this neurotrophin.

### 3.5. Protein Expression Analysis

**Figure 6:**
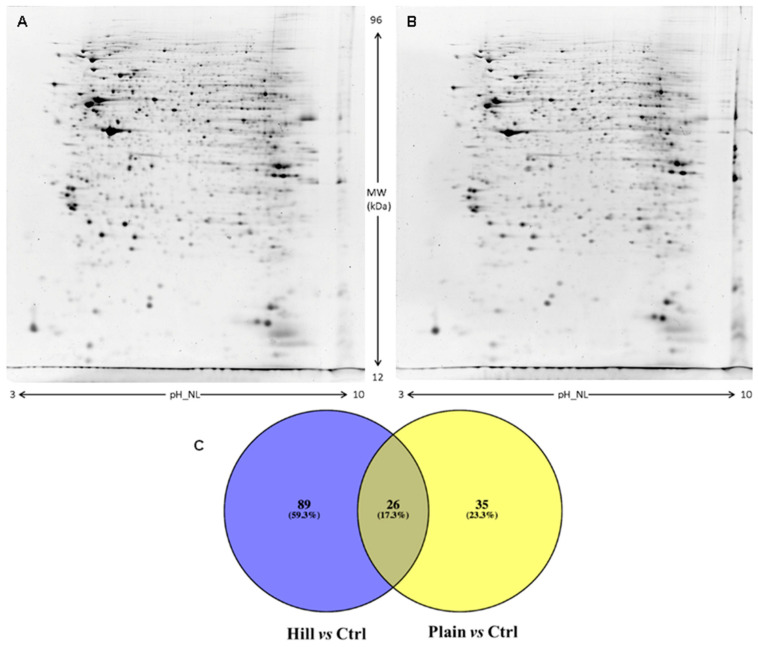
2DE representative images of differentiated SH-SY5Y cell proteins treated with Hill (**A**) and Plain (**B**) extracts. Venn diagram (**C**) of different comparisons. (**A**,**B**) SH-SY5Y proteins were separated in a 3–10 nonlinear gradient. SDS-PAGE was performed using 12% acrylamide. Gels were stained with ruthenium. (**C**) Venn diagram highlighting the distribution of identified differentially expressed proteins in hill and plain extracts as compared to control. Both unique and overlapping proteins are reported as actual number and percentage (Venny 2.0.2).

Figure 6A illustrates a representative two-dimensional electrophoresis (2DE) image of differentiated SH-SY5Y cell proteins. An average of 1220 ± 120 spots was found within a nonlinear pH range from 3 to 10. Comparative analysis was performed between hill or plain extract treated and control cell samples. Venn diagram (Figure 6B) shows the number of common and exclusive proteins between the two comparisons. One-hundred fifteen protein spots were found differentially expressed in hill extract treated cells with comparison to control cells, of which 26 spots were also differentially expressed in plain extract treated cells compared to control cells. Spots, which showed a significant fold change of expression ≥ 1.2 were subsequently subjected to nano-LC-ESI-MS/MS analysis and identified. Table 4 reports the list of identified proteins, which are exclusive of hill extract treated cells together with their MW, pI, peptides and coverage values of MS/MS, ratio and *p* values. A list of common proteins both to hill and plain extract treated cells is shown in Table 5. More than one identification was reported for some spots when MW and pI were not distinguishable.

### 3.6. Bioinformatic Analysis

Using both PANTHER gene classification and Ingenuity Pathway Analysis (IPA) software, we found that the main biological processes, in which the deregulated proteins of hill and plain extract treated SH-SY5Y cells are involved, included “biogenesis,” “metabolic process”, and “cellular process.”

All proteins found differentially expressed in hill extract treated cells were analyzed by IPA to discover the most enriched canonical pathways, possible upstream regulators and downstream effects. The software generated two main networks, “Gene expression, RNA damage and repair, RNA post-transcriptional modifications” and “Cellular development, cellular growth and proliferation, nervous system development and function” with 46 and 32 score values, respectively.

**Figure 7:**
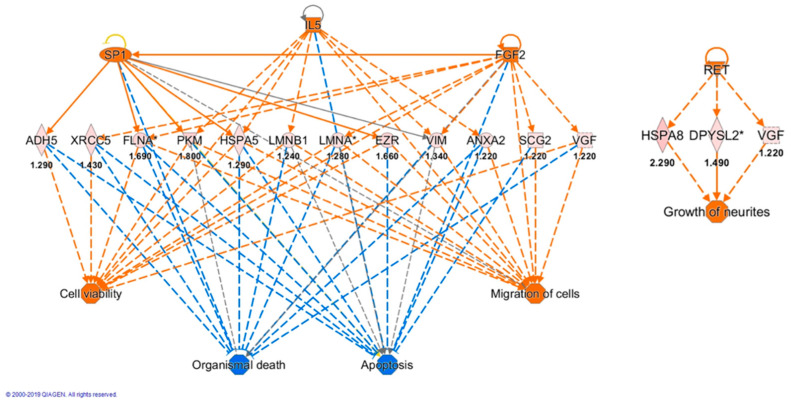
Regulator network generated by IPA. Upstream regulators (SP1, IL5, FGF2 and RET) are displayed in the top tier while functions are displayed in the bottom tier. The data set proteins connecting regulators and lower functions are shown in the middle tier where the fold change value of each protein expression is reported below the gene name. The predicted regulators had a z-score (activation score) > 1.9 and a Fisher’s exact *p*-value < 0.05. Dashed lines are indirect effects and the protein shape indicates the protein class (defined by IPA). Orange and cyan connecting lines indicate activation and inhibition, respectively. Similarly, activated and inhibited downstream functions are orange and cyan. * More than one spot was identified for this protein.

The downstream analysis unveiled key biological and cellular functions such as organismal survival, nervous system development, cell death and survival, gene expression and RNA damage and repair. Moreover, all proteins found differentially expressed concurred in an upstream analysis to predict activation or inhibition of potential transcription factors or molecules. Table 6 shows a list of top upstream regulators based on *p*-value. Moreover, an algorithm connection among upstream regulators, data set molecules and downstream functions or diseases, generated four main regulators with high consistency scores. The following regulators, fibroblast growth factor 2 (FGF2), interleukin-5 (IL5) and transcription factor Sp1 (SP1) revealed that the functional categories most impacted by the presence of the hill EVOO extracts were apoptosis, organismal death, cell viability and migration with consistency score of 13568 (Figure 7). This analysis presumed SP1, IL5 and FGF2 activate 5 to 7 proteins and 2 functions (cell viability and migration) whereas they inhibit organismal death and apoptosis. In addition, the proto-oncogene tyrosine-protein kinase receptor (RET) regulator leads to activation of neurite growth (consistency score = 1732) through increased expression of dihydropyrimidinase-related protein 2 (DPYSL2), heat shock protein A8 (HSPA8) and neurosecretory protein VGF (VGF) (Figure 7). RET regulator also activates RET itself.

### 3.7. Validation of BDNF and DPYSL2 Expression in SH-SY5Y Cells Treated with Hill and Plain EVOO Extracts

**Figure 8:**
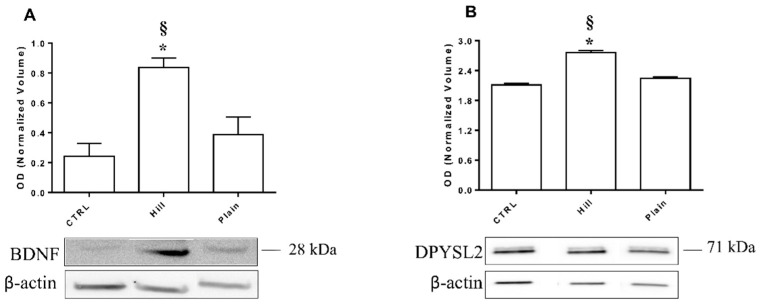
WB analysis of BDNF (**A**) and DPYSL2 (**B**) expression in SH-SY5Y cells treated with and without EVOO extracts. Representative immunoblot images and histograms of band normalized OD are shown. Data are presented as mean ± SEM of three independent experiments. Data were analyzed with a one-way ANOVA followed by the Bonferroni’s test. * *p* < 0.05 vs. CTRL; § *p* < 0.05 vs. plain.

Western blot analysis was used to validate the different expression level of the BDNF protein in hill and plain extract treated cells as suggested by RT-PCR data. Moreover, the different expression level of DPYSL2 was assayed to validate 2DE data. For each tested protein, the optical density of the immunoreactive band was normalized by the optical density of the β-actin immunoreactive band. Representative immunoblots and bar graphs of normalized density values are shown in Figure 8. A single immunoreactive band with apparent molecular weight of approximately 28 kDa and 71 kDa was obtained for BDNF and DPYSL2, respectively. In particular, the protein recognized by the anti-BDNF antibody likely corresponds to unglycosilated pre-pro-BDNF. BDNF and DPYSL2 expression was affected by cell treatment with EVOO extracts, but only the hill extract caused a significant increased expression of BDNF compared both to control and plain extract. On the other hand, both EVOO extracts were able to significantly increase DPYSL2 expression (*p* < 0.001) compared to control.

## 4. Discussion

Quercetano is an endemic cultivar typical of a specific area of Tuscany named “Piana Versiliese”. Quercetano is a millennial tree characterized by small fruits resistant to olive fly thanks to their slow maturation. Due to the characteristics of “Piana Versiliese” Quercetano olive trees grow both in the hill and plain and are influenced by changes in microclimate, soil composition and water availability. Here, we report the characterization of two Quercetano EVOO extracts, hill and plain and their different neuroprotective activity in SH-SY5Y cells.

The different geographical origin of the orchards influenced both the total phenol content and the quantity of single phenols. These results are in agreement with other studies that evidenced how environmental factors such as temperature, soil moisture, water status, light and nitrogen, affect the biosynthesis of phenols and their bioaccumulation [48,49,50,51].

In particular, the plain extract presented a higher content of phenyl ethyl alcohols, cinnamic acids, oleacein (3,4-DHPEA-EDA) and oleocanthal (p-HPEA-EDA) and flavones, meanwhile hill extract was richer in lignans and oleuropein and ligstroside aglycons derivatives (3,4-DHPEA-EA and p-HPEA-EA, respectively). These data are in agreement with the results of Agiomyrgianaki et al. [52], that investigated the composition of EVOO extracts obtained from olives of the same cultivar grown in different geographical areas of Greece. They observed that the total hydroxytyrosol and lignans, pinoresinol and 1-acetoxytyrosol, were the most influential variables in discriminating the Greek olive oil samples according to the different geographical divisions.

The potential antioxidant activity of the extracts has been investigated using the neuroblastoma cell line SH-SY5Y differentiated with retinoic acid. These cells are considered as neuronal precursors and differentiate into more mature neuronal phenotypes under selected growth conditions such as retinoic acid [53,54]. For this reason, they are widely utilized in in vitro studies to dissect out pathogenetic mechanisms of neurodegenerative disorders [55,56].

Of note, the treatment with both extracts increased cell viability of differentiated SH-SY5Y cells. This effect is probably due to an enhancement of the mitochondrial respiration. In fact, MTT assay measures cell viability in terms of reductive activity as enzymatic conversion of the tetrazolium compound to water insoluble formazan crystals by dehydrogenases occurring in the mitochondria of living cells [57]. This hypothesis is supported by the data of Grewal et al. [58], that demonstrated the treatment with ligstroside and, at a lower extent, with 3,4-DHPEA-EDA enhanced mitochondrial respiration in SH-SY5Y-APP695 cells, a cellular model of early AD.

Both extracts showed a good activity in counteracting H_2_O_2_ induced oxidative stress, even if the hill extract was significantly more effective than the plain extract. In particular, the hill extract showed a higher ability to increase cell viability in respect to cells exposed to peroxide and to decrease LDH release. On the other hand, the two extracts had comparable effect in reducing intracellular ROS levels and increasing GSH levels. To better characterize the antioxidant mechanism underpinned by this higher protective activity of the hill extract, we evaluated the expression of four fundamental antioxidant enzymes, namely HMOX1, NQO1, TXNRD1 and GR. HMOX1 catalyzes the degradation of heme to biliverdin, which is subsequently converted to bilirubin by biliverdin reductase and both biliverdin and biliverdin reductase have antioxidant and anti-inflammatory activity [59,60]. NQO1 catalyzes the two-electron reduction of quinones and protects cells from the dangerous effects of semiquinones produced by the one-electron reduction of quinones catalyzed by cytochrome P450 reductase. The semiquinones are known to enter in the redox cycling to generate oxygen free radicals [61]. TXNRD1 is a member of the pyridine nucleotide disulfide oxidoreductase family and catalyzes the reduction of oxidized thioredoxin, using NADPH as the electron donor [62]. Glutathione reductase catalyzes the NADPH-dependent reduction of oxidized glutathione to reduced glutathione and plays a key role in providing adequate levels of reduced GSH [63]. Interestingly, the expression of these antioxidant enzymes is regulated by nuclear factor erythroid 2-related factor 2 (Nrf2) through the binding to the antioxidant response elements (ARE), a characteristic sequence present in the promoter region of antioxidants and phase II enzymes including HMOX1, NQO1, TXNRD1 and GSR [64,65,66]. The hill extract demonstrated a markedly higher ability in boosting up the endogenous antioxidant defense system in respect to the plain extract. Indeed, the hill extract upregulated all four enzymes meanwhile the plain extract just slightly increased the expression of HMOX1 and NQO1. We hypothesize that the different effects of the extracts on the expression of these enzymes could be related to their different phenol composition. The hill extract is characterized by a higher content of the lignans, pinoresinol and acetoxypinoresinol, oleuropein aglycone and ligstroside aglycon. Li et al. [67] showed pinoresinol activates the transcription factor Nrf2 and, in agreement with our data, increases the expression of one of its downstream targets, NQO1. Ligstroside derivatives belong to the secoiridoid class of biophenols, which are molecules exclusively present in plants belonging to the Olearaceae group including *Olea europaea* [68]. In SH-SY5Y-APP695 cells, Grewal et al. [58] demonstrated ligstroside increases the activity of glutathione peroxidase 1, another downstream target of Nrf2 suggesting the higher expression of antioxidant enzymes induced by the hill extract could be associated to a higher presence of compounds able to activate the Nrf2 pathway. Other plant-derived compounds including curcumin, sulforaphane, gamma oryzanol and resveratrol exert neuroprotective and antioxidant activity via Nrf2 [69,70,71,72]. Gamma oryzanol, in particular, could be considered for future studies in comparison to EVOOs since found mainly in rice bran oil.

Moreover, the hill extract, but not the plain extract, significantly increased the expression of BDNF. The neurotrophin BDNF is one of the most studied and well characterized neurotrophic factor in the CNS. BDNF interacts with tropomyosin-related kinase B (TrkB) and p75 cellular receptors and supports maintenance of normal brain function, neurite outgrowth and synaptic plasticity [73,74]. Several lines of evidence indicate BDNF levels play an important role in the pathogenesis of several neurodegenerative diseases, including Alzheimer’s disease (AD) [75]. In the brain of AD patients or aged subjects, BDNF expression levels are lower compared with healthy subjects [76,77]. In vitro, BDNF protects neurons against Aβ_1–42_ and Aβ_25–35_ induced toxicity [78] and promotes the de-phosphorylation of tau protein [79].

Given the higher neuroprotective activity shown by the hill in respect to the plain extract in SH-SY5Y cells, we performed a proteomic analysis to identify further molecular pathways modulated by these extracts. Proteomic data suggested a beneficial effect of both extracts. In fact, both hill and plain extracts induced differential expression of proteins belonging to categories involved in metabolic and cellular processes, biogenesis and cellular component organization and response to stimulus. Particularly, an increase of proteins involved in redox balance, antioxidant defenses (CS, NDUFAF7, UQCRC2) and mitochondrial morphology (IMMT) was observed while cancer promotion proteins (DIABLO, TCPT) decreased. These expression changes agree with our functional results and documented roles of EVOO polyphenols in protecting neuronal cells [80]. Indeed, these polyphenols possess the ability to restore the redox balance and hence, the optimal neuronal function, not only as antioxidants but also as mild pro-oxidants, with ensuing upregulation of cell antioxidant defenses [80]. In consideration of the major proteome changes induced by treating SH-SY5Y cells with the hill extract, we focused our attention on related findings. In cells treated with the hill extract, a large part of the eighty-nine exclusive deregulated proteins belongs to classes of RNA binding, translational and cytoskeletal proteins. Of note, the highest expression increase was observed for proteins, which are involved in cytoplasmic RNA trafficking, pre-mRNA splicing (HNRNPA3, NONO) and neuronal differentiation and maintenance (ELAVL3). IPA enrichment analysis unveiled cellular processes and components affected by the hill EVOO extract. In particular, IPA revealed inhibition of such signaling cascades as those triggered by eNOS, VEGF and ILK suggested the hill extract components were able to induce a deregulation of neuronal cell proteins able to counteract oxidative stress, proliferative and angiogenetic effects potentially promoted by their activation. On the other hand, IPA analysis also revealed activation of Unfolded Protein Response (UPR) and BAG2 pathways, resulting from enhanced transcription of ER chaperones (HSPA1A/HSPA1B, HSPA4, HSPA5, HSPA8), folding enzymes (UBQLN2) and other components of the protein degradation machinery (PSMA2, PSMD2) therefore, suggesting a potential role of the hill extract in reducing the accumulation of misfolded proteins. In addition, IPA upstream regulator analysis pointed out activation of four main regulators (FGF2, IL5, SP1 and RET) with high confidence score. Specifically, FGF2, IL5 and SP1, through twelve target proteins, promote cell viability and migration and, on the other side, inhibit apoptosis and organismal death confirming the protective role of hill extract. In addition, a regulatory role of SP1 for neuronal development and synaptogenesis has been suggested in mice astrocytes [81].

Further to this point, RET regulator activation suggested by overexpression of dihydropyrimidinase-related protein 2 (DPYSL2), neurosecretory protein VGF (non-acronymic) and heat shock cognate 71 kDa protein (HSPA8) induced activation of neurite growth function, also called neuritogenesis. Neuritogenesis is a complex task regulated by an interplay of various neurotrophic factors. Recently, Moutinho et al. [82] have demonstrated that a human VGF-derived antidepressant neuropeptide promotes neurite outgrowth in SH-SY5Y cells in association with BDNF suggesting the important role of these peptides in neuroplasticity linked with learning, memory, depression and chronic pain. Moreover, overexpression of DPYSL2 can induce neural stem cells to differentiate into neurons. In fact, DPYSL2 is considered a novel regulator for neural stem cell differentiation in rats playing a pivotal role in neuronal development and polarity [83]. Similar effects have been also described for the above mentioned ELAVL3. Indeed, Ogawa et al. [84] observed a Elavl3^-/-^ mouse model develops a deficit in axonal transport, abnormalities in neuronal polarity of Purkinje cells and a slowly progressive axonal degeneration. Therefore, the increase of VGF, DPYSL2 and ELAVL3 observed in our cells suggests the hill extract may favor a reprogramming of neuronal cells to activate the neurogenesis machinery which, in turn, may promote a protection from neuronal injury. Concerning neurogenesis, a positive synergic effect of FGF2 and BDNF has been described in dopaminergic motoneurons [85]. In animal models of Parkinson’s disease (PD), BDNF enhances the survival of dopaminergic neurons, improves dopaminergic neurotransmission and motor performance [86]. Moreover, as previously underlined, neurotrophins prevent cell death and support neuronal proliferation and maturation enhancing both growth and function of affected neurons in Alzheimer’s disease (AD) and PD [75,86,87]. Despite the under threshold of BDNF *z*-score value derived by IPA, both transcript and protein expression analysis by PCR and WB confirmed a significant increase of the neurotrophic factor expression in SH-SY5Y cells treated with the hill extract.

Overall, these results suggest a peculiar effect of the hill compared to the plain extract, particularly for triggering the over-expression of key antioxidant enzymes, regulating the expression of proteins involved in neuronal plasticity and potentially activating neurotrophic factors. Both quality and particularity of EVOO depend on several factors [88,89,90] and among them the cultivar is the main. However, we demonstrated that the environmental conditions and soil can deeply affect even the composition of EVOOs obtained from the same cultivar. In respect to the polar polyphenolic composition, the hill extract was characterized by a higher percentage of lignanes and of two secoiridos, namely 3,4-DHPEA-EA and p-HPEA-EA, even if the plain extract contains a higher percentage of total secoiridoids.

Recent evidence suggests a correlation between specific components of extracts and neuronal plasticity. Yu et al. [91] observed that lignan pinoresinol improves memory impairment in a mouse model of dementia influencing the regulatory mechanisms involved in synaptic plasticity, thus making it a promising candidate agent to treat AD. In addition, a neuroprotective and anti-inflammatory activity of pure lignans or lignans extracted from *Viburnum erosum* have been described [92]. Moreover, different studies [58,93,94,95] have described the comparable effects of secoiridoids. In fact, ligstroside-fed mice show improved spatial working memory and enhanced cognitive function [58]. In addition, both in aged and early AD mice, ligstroside expands the lifespan with outstanding performance on mitochondrial bioenergetics [58]. Finally, a role of oleuropein aglycone on α-synuclein aggregation has been reported [94]. Indeed, a recent study has highlighted oleuropein aglycone stabilizes the monomeric α-synuclein and favors the growth of non-toxic aggregates [94]. The evidence reported in literature, which assign lignans and secoiridoids among promising candidates to treat brain disorders, are in line with molecular and function changes observed in SH-SY5Y cells treated with the hill EVOO extract.

## 5. Conclusions

Our data highlight the impact of climatic areas on EVOO phenolic composition and, consequently, how this influences the antioxidant and neuroprotective effects of EVOO extracts. In particular, the hill EVOO extract was more effective in counteracting the oxidative stress and boosting up the endogenous antioxidant system in respect to the plain EVOO extract. Moreover, proteomic analysis revealed that the hill extract also modulates important molecular pathways related to neuronal survival and neurogenesis. Future studies are needed to characterize the role of individual compounds whose content is significantly higher in hill extract, in respect to the plain extract, in the biological activity observed.

## Figures and Tables

**Table 1:** List of primers for real-time PCR in SH-SY5Y cells.

Gene	5′-Forward-3′	5′-Reverse-3′	RefSeq Accession No.
HMOX1	CAACAAAGTGCAAGATTCTG	TGCATTCACATGGCATAAAG	NM_002133
BDNF	CAAAAGTGGAGAACATTTGC	AACTCCAGTCAATAGGTCAG	NM_001143811
NQO1	AGTATCCACAATAGCTGACG	TTTGTGGGTCTGTAGAAATG	NM_000903
GSR	GACCTATTCAACGAGCTTTAC	CAACCACCTTTTCTTCCTTG	NM_000637
TXNRD1	AGACAGTTAAGCATGATTGG	AATTGCCCATAAGCATTCTC	NM_001093771
18S rRNA	CAGAAGGATGTAAAGGATGG	TATTTCTTCTTGGACACACC	NM_022551

**Table 2:** Type of soil in hill and plain orchards ^a^.

Soil Categories	Hill	Plain
	% ± SEM	% ± SEM
Clay	8.7 ± 0.08 *	8.4 ± 0.08
Silt	38 ± 0.46 ***	46.1 ± 0.50
Sandy	53.3 ± 0.68 **	45.6 ± 0.94
pH	5.4 ± 0.04 ***	6.9 ± 0.05
	Ppm ±	Ppm ±
K	110 ± 0.83 ***	54.7 ± 0.44
Mg	73.7 ± 0.47 ***	63.6 ± 0.37
Ca	1188 ± 10.29 ***	1428 ± 15.46
P	48 ± 0.36 ***	34 ± 0.22
Total N	2600 ± 24.02 ***	3000 ± 27.71

^a^ Asterisks after the phenolic substance’s name indicate the level of significance (One way ANOVA, Tukey’s test for pairwise comparison) in the difference between Hill and Plain extracts: * *p* < 0.05; ** *p* < 0.01; *** *p* < 0.001; Analyses were carried out by the “Regional laboratory for soil analysis and plant production” http://www.agriligurianet.it (accessed 21 January 2021).

**Table 3:** Concentration of the main polar phenolic components in hill and plain EVOO’s extracts.

Phenolic Compound	Hill	Plain
	(μg/g ± SD)	
Hydroxytyrosol **	678.21 ± 24.27	2167.85 ± 93.35
Tyrosol **	771.82 ± 38.75	1697.46 ± 123.03
Vanillic acid **	6.83 ± 0.34	4.05 ± 0.15
p-Coumaric acid **	3.22 ± 0.19	10.30 ± 0.67
ferulic acid *	0.56 ± 0.04	0.89 ± 0.05
3,4-DHPEA-EDA **	803.33 ± 46.12	2610.92 ± 169.48
p-HPEA-EDA	1102.60 ± 71.26	1316.25 ± 95.03
Pinoresinol **	1819.41 ± 51.98	664.64 ± 23.78
Acetoxypinoresinol **	1854.62 ± 133.24	1018.40 ± 80.94
Luteolin	64.27 ± 4.15	85.78 ± 6.19
3,4-DHPEA-EA *	330.78 ± 9.45	244.55 ± 8.75
p-HPEA-EA **	756.07 ± 54.32	251.74 ± 20.01
Apigenin *	25.08 ± 1.08	40.08 ± 2.02

Asterisks after the phenolic substance’s name indicate the level of significance (One way ANOVA, Tukey’s test for pairwise comparison) in the difference between Hill and Plain extracts: * *p* < 0.05; ** *p* < 0.01; 3,4-DHPEA-EDA: dialdehydic form of decarboxymethyl elenolic acid linked to hydroxytyrosol (oleacein); p-HPEA-EDA: dialdehydic form of decarboxymethylelenolic acid linked to tyrosol (oleocanthal); 3,4-DHPEA-EA: isomer of oleuropein aglycone; p-HPEA-EA: ligstroside aglycon.

**Table 4:** List of differentially expressed proteins, which are exclusive of hill extract treated cells.

Spot n.	Protein Name	ID	Gene	Coverage (%)	Peptides	Unic Peptide	MW (kDa)	pI	Ratio (Hill/Ctrl)	*p*-Value
2417	Heterogeneous nuclear ribonucleoprotein A3	P51991	HNRNPA3	29	10	10	39	9.1	7.84	0.017
2514	ELAV-like protein 3, Iso 1,2	Q14576	ELAVL3	11, 11	3	1	39/38	9.3	7.3	0.012
1601	Non-POU domain-containing octamer-binding protein	Q15233	NONO	14	4	4	54	9.0	4.59	0.03
1030	Pyruvate dehydrogenase phosphatase regulatory subunit, mitochondrial, Iso 1	Q8NCN5	PDPR	4	3	3	99	5.7	2.48	0.017
1278	Heat shock 70 kDa protein 1A, Iso 1,2	P0DMV8	HSPA1A	13	6	1	66/70	5.4/5.5	2.3	0.011
1278	Heat shock 70 kDa protein 1B	P0DMV9	HSPA1B	13	6	1	70	5.4	2.3	0.011
1278	Heat shock cognate 71 kDa protein	P11142	HSPA8	34	18	14	71	5.3	2.29	0.011
1367	Dihydropyrimidinase-related protein 3, Iso LCRMP-4	Q14195	DPYSL3	28	14	14	74	5.9	2.20	0.022
1606	Pyruvate kinase PKM, Iso M2	P14618	PKM	38	18	18	58	7.9	1.8	0.034
1085	Membrane primary amine oxidase, Iso 1,2	Q16853	AOC3	3	2	2	84/70	6.0/7.1	1.8	0.023
1085	Vitamin D-binding protein, Iso 1,3	P02774	GC	8	2	2	53/55	5.1/5.4	1.77	0.023
894	Filamin-A, Iso 1,2	P21333	FLNA	4	7	7	280	5.7	1.69	0.016
1136	Ezrin	P15311	EZR	10	5	2	69	5.9	1.66	0.005
895	Filamin-A, Iso 1,2	P21333	FLNA	3	6	6	280	5.7	1.54	0.01
1093	ATP-dependent 6-phosphofructokinase, muscle type, Iso 1, 3	P08237	PFKM	15	9	9	85/93	8.2	1.50	0.005
945	Prolyl 3-hydroxylase 3	Q8IVL6	P3H3	6	4	4	82	5.8	1.50	0.008
1427	Dihydropyrimidinase-related protein 2, Iso 1,2	Q16555	DPYSL2	15/16	5	5	62/58	5.9/5.7	1.49	0.033
2475	Eukaryotic translation initiation factor 2 subunit 1	P05198	EIF2S1	30	7	7	36	5.0	1.46	0.029
569	Hypoxia up-regulated protein 1	Q9Y4L1	HYOU1	45	32	32	111	5.1	1.45	0.014
781	Heat shock 70 kDa protein 4	P34932	HSPA4	32	18	18	94	5.1	1.39	0.044
1485	Very long-chain specific acyl-CoA dehydrogenase, mitochondrial, Iso 1,2,3	P49748	ACADVL	4	2	2	70/68	7.7/8.7	1.38	0.001
1280	Heterogeneous nuclear ribonucleoprotein M, Iso 1,2	P52272	HNRNPM	18	8	8	77/74	8.8/ 8.9	1.36	0.019
1371	Probable ATP-dependent RNA helicase DDX17, Iso 1,2,3,4	Q92841	DDX17	8	4	4	80/72	8.5/ 8.8	1.36	0.019
1371	Calcium-binding mitochondrial carrier protein Aralar2, Iso 1,2	Q9UJS0	SLC25A13	17	8	8	74	8.7	1.36	0.019
2074	Vimentin	P08670	VIM	27	10	10	53	5.0	1.34	0.004
952	26S proteasome non-ATPase regulatory subunit 2	Q13200	PSMD2	19	10	10	100	5.1	1.34	0.017
808	Insulin-degrading enzyme	P14735	IDE	3	3	3	117	6.2	1.33	0.043
1216	78 kDa glucose-regulated protein	P11021	HSPA5	46	30	30	72	5.0	1.29	0.003
2374	Alcohol dehydrogenase class-3	P11766	ADH5	18	4	4	39	7.6	1.29	0.037
2417	Heterogeneous nuclear ribonucleoproteins A2/B1, Iso B1	P22626	HNRNPA2B1	12	3	3	37	8.9	1.29	0.037
1413	Prelamin-A/C, Iso A,C	P02545	LMNA	9	5	5	74/65	6.5/6.4	1.28	0.023
1650	Non-POU domain-containing octamer-binding protein	Q15233	NONO	25	9	9	54	9.0	1.28	0.041
901	Programmed cell death 6-interacting protein, Iso 1,2	Q8WUM4	PDCD6IP	21	11	11	96		1.28	0.006
1371	Heterogeneous nuclear ribonucleoprotein M, Iso 1,2	P52272	HNRNPM	16/17	8	8	77/75	8.8/8.9	1.27	0.023
1461	Asparagine--tRNA ligase, cytoplasmic	O43776	NARS	31	13	13	63	5.9	1.26	0.037
1467	Prelamin-A/C, Iso A,C	P02545	LMNA	16/19	9	9	74/65	6.5/6.4	1.26	0.043
811	Neutral alpha-glucosidase AB, Iso 1,2	Q14697	GANAB	10	8	8	106/109		1.26	0.001
1012	Gelsolin, Iso 1,2,3,4	P06396	GSN	6	3	3	85/80	5.7/5.5	1.24	0.041
1363	Lamin-B1	P20700	LMNB1	33	20	20	66	5.1	1.24	0.03
1330	Beta-catenin-like protein 1, Iso 1,4	Q8WYA6	CTNNBL1	7	2	2	65/61	4.9/5.0	1.24	0.03
1330	Ubiquilin-2	Q9UHD9	UBQLN2	5	2	2	65	5.1	1.24	0.03
1084	Neurosecretory protein VGF	O15240	VGF	14	6	6	67	4.7	1.22	0.013
1084	Secretogranin-2	P13521	SCG2	14	7	7	71	4.6	1.22	0.013
2593	Elongation factor 1-delta, Iso 1,2	P29692	EEF1D	26	6	6	31	4.9/6.0	1.16	0.015
1427	Phosphoacetylglucosamine mutase, Iso 1,2,3	O95394	PGM3	6	3	3	59/62	5.8/5.6	1.07	0.023
3410	Proteasome subunit alpha type-2	P25787	PSMA2	39	7	7	26	7.1	0.81	0.002
2315	Actin, cytoplasmic 1	P60709	ACTB	11	4	4	42	5.2	0.75	0.014
2315	Actin, cytoplasmic 2	P63261	ACTG1	11	4	4	42	5.3	0.75	0.014
1979	Ribonuclease inhibitor	P13489	RNH1	19	6	6	50	4.7	0.73	0.017
2230	DNA-directed RNA polymerases I and III subunit RPAC1, Iso 1,2	O15160	POLR1C	17/16	4	4	39/38	5.3/5.6	0.67	0.007
2830	Voltage-dependent anion-selective channel protein 2, Iso 1,2,3	P45880	VDAC2	15/16	3	3	33/30	7.5/6.8	0.66	0.023

**Table 5:** List of common differentially expressed proteins.

Spot n.	Protein Name	ID	Gene	Coverage (%)	Peptides	Unic Peptides	MW (kDa)	pI	Ratio (Plain/Ctrl)	Ratio (Hill/Ctrl)	*p*-Value
2164	Heterogeneous nuclear ribonucleoprotein D0 Iso 1,3	Q14103	HNRNPD	9/10	2	2	38/32	7.6/8.2	1.77	1.44	0.005
2164	Citrate synthase mitochondrial	O75390	CS	8	3	3	52	7.4	1.77	1.44	0.005
2164	Protein arginine methyltransferase NDUFAF7 mitochondrial	Q7L592	NDUFAF7	8	3	3	49	7.3	1.77	1.44	0.005
2164	Cytochrome b-c1 complex subunit 2. mitochondrial	P22695	UQCRC2	14	4	4	48	7.7	1.77	1.44	0.005
1001	Pre-mRNA-splicing factor ATP-dependent RNA helicase DHX15	O43143	DHX15	14	9	9	91	7.1	1.72	1.49	0.009
5503	MICOS complex subunit MIC60, Iso 1,2, 4	Q16891	IMMT	31/31	17	1	83/ 82	5.7/6.1	1.66	1.75	0.019
1057	X-ray repair cross-complementing protein 5	P13010	XRCC5	13	7	7	83	5.5	1.62	1.43	0.0006
1993	26S proteasome regulatory subunit 7	P35998	PSMC2	38	14	14	48	5.7	1.58	1.23	0.005
881	Elongation factor 2	P13639	EEF2	9	6	6	95	6.4	1.58	1.29	0.033
726	Vinculin, Iso 1,2	P18206	VCL	16/15	9	9	116/124	5.8/5.5	1.38	1.63	0.028
2654	Annexin A2, Iso 1,2	P07355	ANXA2	46/44	18	18	38/40	7.5/ 8.5	1.24	1.22	0.036
3761	Adenine phosphoribosyltransferase, Iso 1,2	P07741	APRT	23/31	3	3	19/14	5.7/6.7	0.87	0.81	0.036
3761	DNA-directed RNA polymerase II subunit RPB7	P62487	POLR2G	23	3	3	19	5.3	0.87	0.81	0.036
3549	Eukaryotic translation initiation factor 3 subunit K, Iso 1,2	Q9UBQ5	EIF3K	11	2	2	25/24	4.8. 4.7	0.7	0.73	0.013
3549	Proteasome subunit beta type-6	P28072	PSMB6	18	4	4	25	4.9	0.7	0.73	0.013
3549	Translationally-controlled tumor protein	P13693	TPT1	34	5	5	19	4.8	0.7	0.73	0.013
3977	Diablo homolog. Mitochondrial, Iso 1,2	Q9NR28	DIABLO	27/34	6	6	27/21	4.7/4.8	0.58	0.52	0.003
3977	Ras-related protein Rap-2a	P10114	RAP2A	10	2	2	20	4.73	0.58	0.52	0.003

**Table 6:** List of top eight upstream regulators obtained by IPA analysis of differentially expressed proteins in hill extract treated SH-SY5Y cells.

Upstream Regulator	Molecule Type	Activation z-Score	*p*-Value of Overlap	Target Molecules
TP53	Transcription regulator	0.295	8.48 × 10^−11^	ACADVL, ACTB, ADH5, ANXA2, CS, EZR, GC, GSN, HNRNPA2B1, HSPA1A/HSPA1B, HSPA8, NARS1, PDCD6IP, PFKM, PGM3, PKM, PSMA2, PSMD2, RAP2A, SLC25A13, UBQLN2, VCL, VIM, XRCC5
MAPT	Other		7.74 × 10^−9^	ACTB, ACTG1, CS, DPYSL2, DPYSL3, EEF2, HSPA1A/HSPA1B, HSPA5, HSPA8, PKM, PSMD2
BDNF	Growth factor	0.29	2.53 × 10^−7^	ANXA2, DPYSL3, EEF1D, EEF2, FLNA, HSPA5, RNH1, VGF, VIM
IL5	Cytokine	2.236	1.25 × 10^−3^	ANXA2, HSPA5, LMNB1, PKM, VIM
SP1	Transcription regulator	2.211	5.07 × 10^−3^	ADH5, EZR, FLNA, HSPA5, PKM, VIM
FGF2	Growth factor	2.391	1.1 × 10^−3^	FLNA, LMNA, SCG2, VGF, VIM, XRCC5
RET	Kinase	2.186	1.6 × 10^−5^	DPYSL2, HSPA1A/HSPA1B, HSPA8, UBQLN2, VGF
MYC	Transcription regulator	2.185	1.32 × 10^−5^	ACTB, EEF2, EIF2S1, EZR, FLNA, HNRNPA2B1, HNRNPD, NARS1, PFKM, PKM, POLR2G, VDAC2, VIM

## Data Availability

The data used to support the findings of this study are available upon request to the authors.
